# Regulation of Human Cytochrome P4501A1 (hCYP1A1): A Plausible Target for Chemoprevention?

**DOI:** 10.1155/2016/5341081

**Published:** 2016-12-26

**Authors:** Rebeca Santes-Palacios, Diego Ornelas-Ayala, Noel Cabañas, Ana Marroquín-Pérez, Alexis Hernández-Magaña, Sitlali del Rosario Olguín-Reyes, Rafael Camacho-Carranza, Jesús Javier Espinosa-Aguirre

**Affiliations:** Departamento de Medicina Genómica y Toxicología Ambiental, Instituto de Investigaciones Biomédicas, UNAM, Av. Universidad 3000, Col. Ciudad Universitaria, 04510 Ciudad de México, Mexico

## Abstract

Human cytochrome P450 1A1 (hCYP1A1) has been an object of study due to its role in precarcinogen metabolism; for this reason it is relevant to know more in depth the mechanisms that rule out its expression and activity, which make this enzyme a target for the development of novel chemiopreventive agents. The aim of this work is to review the origin, regulation, and structural and functional characteristics of CYP1A1 letting us understand its role in the bioactivation of precarcinogen and the consequences of its modulation in other physiological processes, as well as guide us in the study of this important protein.

## 1. Introduction

Cytochrome P450 (CYP) is a superfamily of hemoproteins, with monooxygenase activity, which are spread into the three domains of life. They are biological catalysts that metabolize endogenous compounds such as hormones, bile acids, cholesterol, and xenobiotics like environmental pollutants and drugs. The hCYP1A1 is an enzyme of biomedical and toxicological interest, which catalyzes the biotransformation of polycyclic aryl hydrocarbons (PAHs), aromatic amines, and polychlorinated biphenyls into polar compounds, which can be conjugated to soluble compounds suitable for excretion by urine or bile. Nevertheless, under specific circumstances, this enzyme catalyzes the bioactivation of compounds capable of reacting with macromolecules, such as DNA, leading to the start of mutagenic process.

Every day, we are exposed to compounds that are substrates of CYP1A1, through environmental pollution, food, and, particularly, cigarette smoke. The importance of this protein in chemical carcinogenesis induced by PAHs has been demonstrated in* CYP1* knockout mice, in which the lack of this protein shows less formation of adducts PAH-DNA [1, 2]. In addition, rodent exposition to CYP1A1 inhibitors diminished the number of tumors induced by PAHs [3, 4].

Epidemiologic studies focused on the relationship among PAH exposition, PAH-DNA adducts level, and cancer incidence in humans demonstrate an increased risk in colon adenocarcinoma [5], breast cancer [6], and lung cancer [7] in those individuals with higher levels of adducts.

This data suggests that imbalance between detoxification and bioactivation of carcinogens, independence of enzyme catalysis, regulation of gene expression of* CYP1A1*, and cellular environment are crucial factors at the beginning of chemical carcinogenesis process. Because of this, several questions are still to answer; we propose that a global view of the function and regulation of this enzyme would help to answer these questions; thus, the aim of this work is to integrate the knowledge that has been generated until now about the origin, regulation, and structural characteristics of hCYP1A1.

## 2. Some Aspects of CYP1A1 Evolution

CYPs constitute a superfamily of ancient genes encoding to heme-thiolate proteins that catalyze the monooxygenation of endogenous and exogenous substrates in bacteria, archaea, eukaryotes, and viruses [8, 9]; therefore these proteins must descend from a prokaryotic common ancestor ~3 billion years ago, before the oxygenation of the atmosphere and emergence of eukaryotic cells [10, 11].

The first CYP proteins were involved in the biosynthesis of compounds required for the formation and maintenance of cell structures and then following CYP proteins coevolved as defense mechanisms in plants and insects and more recently a set of these enzymes evolved in response to xenobiotics [12, 13].

CYPs belonging to families 1–4 are the main mediators of exogenous metabolism; however, cytochromes from family 1 are of particular biomedical and toxicological interest because of their affinity to halogenated polycyclic, aromatic amines, aromatic hydrocarbons, and endogenous compounds, whose metabolites can be toxic, mutagenic, or carcinogenic [14–16].

CYP genes of family 1 are grouped into six subfamilies: CYP1A, CYP1B, CYP1C, CYP1D, CYP1E, and CYP1F, from these 1E and 1F are found in urochordates; 1A, 1B, 1C, and 1D are found in fish and amphibians; in mammals the subfamilies that are mainly distributed are 1A and 1B and in some cases 1D [9, 17].

**Figure 1:**
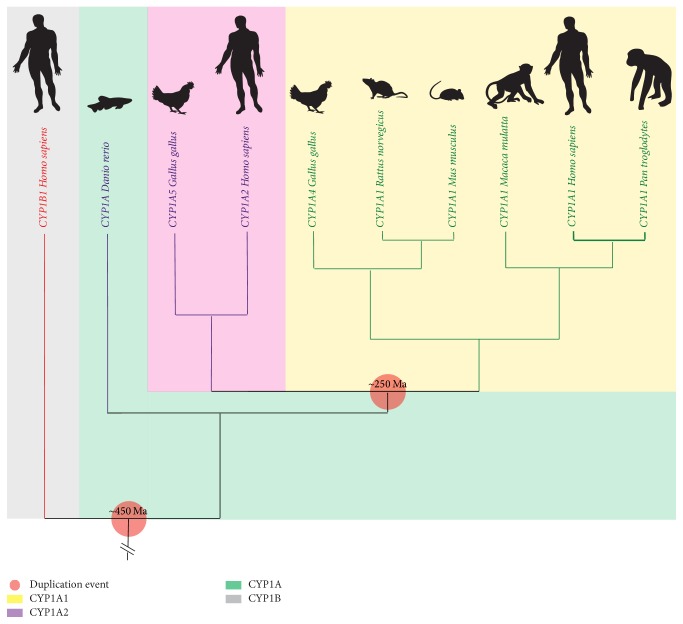
Phylogenetic tree of CYP1A subfamily through different species to human. Amino acid sequences and accession numbers of different species CYP were obtained from the Uniprot database, and with them phylogenetic tree was built in* phyloT: a tree generator* and visualized with* ITOL v3 Interactive Tree Of Life*. Silhouettes, background colors, and symbols were added to the image using Adobe Illustrator CC 2015.0.0 program.

CYP1A and CYP1B diverged from a common ancestor ~450 million years ago (Ma); thus, CYP1A appears early in aquatic vertebrates, as a single copy, which has been identified in teleost fish, while mammals and birds have paralogous genes of CYP1A: CYP1A1, CYP1A2, in mammals, and CYP1A4, CYP1A5 in birds, which emerged ~250 Ma from a duplication event and one inversion, common for both lineages [15, 18, 19] (Figure 1).

In humans, the CYP1A1 gene consists of 6069 bp and is located at the CYP1A1_CYP1A2 locus on chromosome 15q24.1, sharing a regulatory region of 23306 bp with the CYP1A2 gene that is oriented in opposite direction. The 5′ flanking region is shared by both genes and contains a bidirectional promoter and DNA motifs, known as response elements, that activate and regulate the expression of these genes [20, 21].

The participation of multiple signaling pathways in the regulation of the hCYP1A1 transcription has been reported. Next, an overview about the pathways involved in this regulation is reviewed.

## 3. Upregulation of CYP1A1

The constitutive* hCYP1A1* gene has low level of expression in extrahepatic tissues of adult humans. However, liver and extrahepatic expression of this enzyme can be induced by many substrates through multiple pathways. The aryl hydrocarbon receptor (AHR) pathway has been widely studied and it appears to be the main protein receptor that influences* CYP1A1* induction. The AHR is a cytosolic ligand-activated transcription factor associated with two heat shock proteins of 90 kDa (Hsp90), a hepatitis B virus X-associated protein (XAP2), and a chaperone of 23 kDa (p23). This receptor is activated by endogenous ligands and several xenobiotics such as polycyclic aromatic hydrocarbons (PAHs), heterocyclic amines, and halogenated biphenyls [22]. After ligand activation, AHR undergoes conformational changes that promote its translocation into the nucleus, via *β* importin, where it is dissociated from the chaperone proteins (Hsp90, XAP2, and p23), and binds to the nuclear translocator AHR (ARNT) [23, 24]; then the formed AHR-ARNT complex (AHRC) binds to xenobiotic responsive elements (XRE) (5′-TNGCGTG-3′) located at the enhancer element [25].

**Figure 2:**
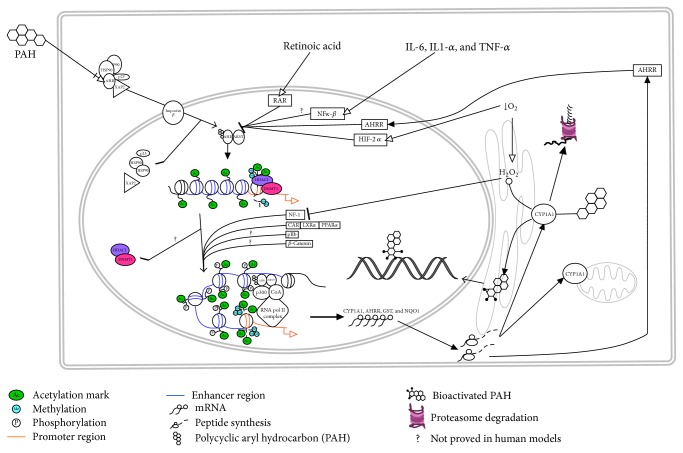
Mechanisms involved in the CYP1A1 regulation. Pathways implicated in up- and downregulation of CYP1A1 are shown, as well as changes in epigenetic marks upon the induction of this gene. The “?” symbol means pathways that had not been proved in human models, specified along the text. Image was created using PathVisio program [42] and edited with Adobe Illustrator CC 2015.0.0 program.

Thirteen XRE have been identified in the regulatory region of human* CYP1A1* [25]. It has been speculated that they are located at the major grooves of the DNA and they would be exposed during nucleosomal movements, allowing the AHRC binding. In turn, this promotes the recruitment of chromatin remodeling proteins such as p300, SRC1/2, and BRG1 [26], subsequent hyperacetylation of lysines 9 and 14 in histone 3 (H3K9ac and H3K14ac), and methylation of lysine 4 in histone 3 (H3K4me) (from dimethylation to trimethylation) at the promoter; meanwhile hyperacetylation of lysine 16 in histone 4 (AcH4K16) and increased phosphorylation of serine 10 in histone 3 (pH3S10) take place at the enhancer element. The increase of acetylation marks at the promoter region of mouse CYP1A1 (mCYP1A1) is consistent with the releasing of a basal repressive complex, which is composed of histone deacetylase 1 (HDAC1) and DNA methyltransferase 1 (DNMT1). It has been suggested that marks at the enhancer could stabilize the open chromatin state to allow the AHRC-mediated transcriptional loop [27–29]. Finally, this AHR-dependent pathway has target genes such as* CYP1A1*,* CYP1A2*, and* CYP1B1* and aldehyde dehydrogenase 3A1 (*ALDH3A1*) [30, 31].  Figure 2 shows some regulatory mechanisms involved in* CYP1A1* regulation.

A number of pathways also modulate* CYP1A1* transcription through binding to the promoter, interactions with AHR, or both mechanisms. Next, we briefly describe some of them.

The canonical Wnt/*β*-catenin signaling pathway is involved in the adult tissue homeostasis regulation, embryonic development, and tumorigenesis. It has also been implicated in the induction of some CYPs, including m*Cyp1a1*. In mice, this was demonstrated by the specific loss of* CTNNB1* that encodes *β*-catenin and leads to a decrease of* mCyp1a1* induction by AHR agonists such as 3-methylcholanthrene (3-MC), *β*-naphthoflavone (*β*-NF), and butylated hydroxyanisole. Additionally, it has been observed that maximum* mCyp1a1* induction was obtained when *β*-catenin acted as coactivator of AHR, although this protein also binds to the transcription factor TCF, which has a binding site in* mCyp1a1* promoter, suggesting a different mode of action [32–34]. Similarly, in rat hepatoma, it has been observed that the interaction between AHR and hypophosphorylated retinoblastoma protein (pRb) aids maximum induction of rat* CYP1A1* by 2.3, 7.8 tetrachlorodibenzo-p-dioxin (TCDD); pRb plays an important role in cell cycle control and it has been proposed that it could also act as a coactivator of AHR [35, 36].

Furthermore, several nuclear receptors are involved in the upregulation of* hCYP1A1*; for example, the constitutive androstane receptor (CAR) [37] which is also a regulator of the expression of the CYP2A, 2B, 2C, and 3A subfamilies is activated by drugs; the liver X receptor *α* (LXR*α*) that is involved in lipid homeostasis is activated by oxysterols [38, 39]; and the peroxisome proliferator-activated receptor *α* (PPAR*α*), is activated by fibrates, phthalates, arachidonic acid, and its derivatives [40, 41]. These receptors bind to their specific responsive elements located in the gene promoter, activate the transcription, and potentiate the induction of* hCYP1A1*. The crosstalk amongst signaling pathways involved in regulating the expression of* CYP1A1* could have implications for drug-drug, drug-toxic, and drug-food interactions.

## 4. Downregulation of CYP1A1

The tight regulation of* CYP1A1* is highly necessary due to the known harmful effects of electrophilic compounds produced by the enzymatic activity of CYP1A1; a number of CYP1A1 downregulation mechanisms have been described; for example, the AHR repressor protein (AHRR) is a target gen of the transcriptional activity of AHR and competes with AHR for binding to XREs. AHRR has been described as a negative tissue-specific regulator of* mCYP1A1* expression [43, 44]. Its overexpression in transgenic mice suppresses the* mCYP1A1* induction in lung, spleen, and adipose tissue [45]. Moreover, it has been suggested that rat CYP1A1 regulates its own expression because it catalyzes the removal of AHR agonists and thus decreases the activation of this pathway [46, 47].

Hypoxia inducible factor participates as a negative regulator of* hCYP1A1* expression through the competition with AHR for the binding to ARNT. Under hypoxia conditions, basal* hCYP1A1* expression decreases [48] and induction by AHR ligands is inhibited [49, 50].

Moreover, the retinoic acid receptor pathway (RAR) is also implicated in the regulation of* hCYP1A1* expression through two mechanisms. In the first one, RAR modulates the transcriptional expression of this protein through its binding to a retinoic acid responsive element (RARE) located in the* hCYP1A1* promoter [51, 52]. In the second one, the corepressor SMRT (silencing mediator for retinoid and thyroid receptors), which is attached to RAR, is released upon activation of RAR by retinoic acid; subsequently released SMRT can interact with AHR and reduce* hCYP1A1* induction [53].

Another protein involved in the downregulation of* hCYP1A1* induction is the nuclear factor I (NFI). NFI activates the expression by binding to promoter of* hCYP1A1* and it is sensitive to oxidative stress [54]. It has been demonstrated that increased activity of hCYP1A1 generates reactive oxygen species, which in turn can lead to the oxidation of the single cysteine residue on NFI and then it is released from the* hCYP1A1* promoter, thus decreasing the expression of this gene [55, 56].

The presence of a glucocorticoid responsive element in the intron one of the* CYP1A1* gene in several species has been reported. The activity of the glucocorticoid receptor potentiates the effect of activated AHR in rat hepatocytes unlike human hepatocytes where dexamethasone (glucocorticoid analog) decreases the* hCYP1A1* protein but not mRNA induced by 3-MC [57, 58]. However, additional studies are needed to clarify the effect of glucocorticoids on* CYP1A1* gene and protein levels.

Gut-enriched Kruppel like factor (KLFG or KLF4) is a regulator of cell proliferation, differentiation, apoptosis, and cellular reprogramming and has been identified as a negative regulator of rat* CYP1A1* transcription in a dependent way of its binding to the basic transcription element (BTE); moreover, this effect might also be part of the interaction between KLFG and Sp1, an* CYP1A1* transcriptional activator [59].

Another kind of downregulation is through the action of proinflammatory cytokines IL-1*β* and IL-6, TNF-*α*, and lipopolysaccharides; these cytokines decrease constitutive CYP1A1 expression and AHR-mediated induction in human and mouse hepatocytes [60–64].

## 5. Epigenetic, Posttranscriptional, and Posttranslational Regulation of CYP1A1

Until now, several modes of action have been reported for the regulation of human CYP1A1. In essence, transcriptional expression has been reviewed, but there is another kind of gene regulation that involves epigenetic mechanisms such as methylation, acetylation, histone ubiquitination, or DNA methylation and hydroxylation. In this regard, to explore the role of these mechanisms on the regulation of* hCYP1A1* expression studies were conducted using the DNMTs inhibitor, 5-aza-2-deoxycytidine (5AzadC), and HDACs inhibitors, trichostatin A (TSA) and sodium butyrate.  Table 1 summarizes the effects of these inhibitors on* CYP1A1* expression. Such effects are species-specific and depend on whether the tissue is derived from healthy or cancerous donations. This review focuses mainly on hCYP1A1 regulation and just on enriching the data presented; Table 1 shows results from studies conducted in human, mouse, or rat cell lines primary cultures.

According to the results it is not possible to conclude whether* hCYP1A1* has a DNA methylation dependence regulation or not. It seems that tissue and temporal issues might have been involved in this regard as well as the tumor state. We cannot rule this, but tumor or cancer state allows an increased DNA methylation in* hCYP1A1* regulatory region, at least in prostate [27] and lung [65, 69]; thus, in these models this gene has no constitutive expression which is activated by exposition to 5AzadC.

There is another type of* hCYP1A1* regulation, which is through posttranscriptional modulation. Some in silico studies have been conducted in order to determine a possible regulation of CYP1A through noncoding RNAs. Based on web databases analyses, six putative micro RNAs (miRNAs), hsa-miR-125b-2, hsa-miR-488, hsa-miR-657, hsa-miR-892a, hsa-miR-511, and hsa-miR-626, with one or more binding sites to the 3′UTR region of* hCYP1A1* were identified [21]. Following the same strategy, an additional study used five different bioinformatics programs and predicted 332 miRNAs to target* hCYP1A1* UTRs, from which 12% were predicted in at least 2 programs [110].

Interestingly, in a study performed in human breast cancer cell line MCF-7 exposed to BaP leads to diminish miR-892a expression and function. This miRNA binds to 515–535 nucleotides of 3′-UTR of human* CYP1A1* and acts as translational repressor of this transcript. The putative effect of miR-892a was previously predicted by an in silico study [111]. Another study conducted in normal human liver tissues (*n* = 92) searched for a correlation between the protein level of CYP1A1 and the expression of miRs and a negative correlation was found for miR-200a (*r*_*s*_ = −0.36), miR-142-3p (*r*_*s*_ = −0.36), and miR-200b (*r*_*s*_ = −0.36) [112]. Nevertheless, another study with healthy human liver tissues from individuals of different ages determined that upregulation of miR-125b-5p was related to downregulation of CYP1A1 from fetal and pediatric samples. The effect of this miRNA was also previously predicted [113].

At this point we realize that the protein expression of CYP1A1 is tissue-, health- and age-specific; thus, it is not strange to expect that also the mechanisms and factors involved in its expression would be specific as we can observe from the previous data where two miRNAs were predicted in silico and confirmed in vivo, but none of them were found repeatedly among the studies reviewed here. It would be obvious that if there are differences in miRNAs found among results with human CYP1A1, there could be much more differences between human and other species models. This assumption is supported by a report conducted in mice fetal thymocytes where miR-31 was found as a negative regulator of* mCyp1a1* translation after exposition of cells to TCDD. Furthermore, miR-31 has matched with 3′-UTR of the transcript of this protein [114].

There are some studies reporting indirect regulation of CYP1A1 through the regulation of AHR by small noncoding RNAs, as in the case of the Sprague-Dawley rats treated during 2 weeks with an antagonist of the corticotrophin releasing factor I. Results show that rat liver CYP1A1 expression was increased through an atypical pathway different from AHR ligand and suggest the involvement of miR-29a-5p, miR-680, and miR-700 which were negatively expressed 10-, 6- and 8.6-fold, respectively. Whether these miRNAs could act through rCYP1A1 direct binding or not is still unknown because the first two had binding sites in the 3′-UTR region of both rCYP1A1 and AHR [115]. More information about hCYP1A1 regulation through its 3′UTR region shall be discovered in the near future to achieve this objective; also more tissues and health conditions are needed to be studied.

Until this point we covered evolutionary origin of CYP1A1 and its transcriptional and posttranscriptional regulation, but once the CYP1A1 protein is formed its cellular lifetime is regulated too. The half-life time of this protein is of ~2.8 hours; this suggests a mechanism of protein degradation and the studies prompted to proteasomal degradation pathway. In fact, treatment with ubiquitin-proteasome inhibitor MG132 keeps the levels of CYP1A1, while lysosomal inhibitors do not [116–118]. In spite of these experiments, there are no reports that could help us figure out the mechanism of degradation of CYP1A1.

Another possible regulation of CYP1A1 is through the degradation of its heme group, which has been explored in human hepatoma cell line HepG2 exposed to different heavy metals. Here an increase in hemooxygenase 1 was found; this enzyme is involved in the metabolism of the heme group. Its increased levels found after heavy metals exposition correlate with diminished activity of CYP1A1, while protein level and gene expression remain unchanged [117, 119, 120].

## 6. Structural Characteristics of Human CYP1A1 and Its Ligands

Human CYP1A1 has a molecular weight of 58.16 kDa and consists of 512 amino acids of which the first thirty of the N-terminal region allow the association of the protein with the mitochondrial membrane and the disordered region of the smooth endoplasmic reticulum rich in unsaturated fatty acids, unlike the human CYP1A2 which is located in the sorted regions rich in cholesterol, sphingomyelin, and saturated fatty acids. Moreover, these thirty residues would also be mediating the interaction with NADPH-CYP reductase [121–124].

Directed mutagenesis in the residues of the human protein showed altered kinetic parameters and demonstrates the importance of certain amino acids like Phe123, Phe224, Glu256, Asp313, Gly316, Ala317, Thr321, Val382, and Ile386 (Table 2) in the recognition, binding, and affinity for the substrates. However, the spatial orientation of these residues was known until the three-dimensional structure of human CYP1A1 was resolved by X-ray crystallography at a resolution of 2.6 Å [125].

**Figure 3:**
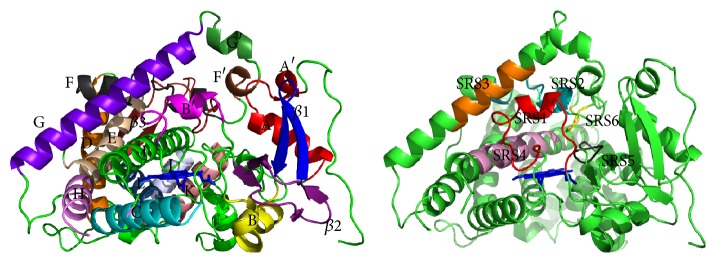
Three-dimensional structure and substrate recognition sites (SRS) of human CYP1A1. Figure was created with PyMOL Molecular Graphics System, Version 1.3 Schrödinger, LLC.

The protein crystallization of human CYP1A1 allowed us to know that this protein is comprised by twelve *α*-helices (A–L), three *β*-sheets (*β*1–*β*3), and four helical short regions (A′, B′, F′, and G′) forming six sequences as putative substrate recognition sites (SRS) important for ligand selectivity of this enzyme [125, 126], which are shown in Figure 3 and listed as follows.

- SRS1 corresponds to the amino acid region 106–124 of loop between helix B and helix B′ and portion of loop between helix B′ and helix C. In turn, it forms part of the wall of the active site and it is proposed as a site for the input and output of ligands that influence the regioselectivity for the oxidation of substrates [127, 128].
- SRS2 is part of the helices E and F, as well as of the residues 217–228, in the loop that connects these regions. Its role is similar to SRS1 participating in the ligand orientation [129, 130].
- SRS3 is found in helix G from amino acid 251 to amino acid 262 [126].
- SRS4 corresponds to helix I (residues 309–324) [126].
- SRS5 goes from residue 381 to residue 386 and connects helix J to the beta sheet. In other CYPs this region has been associated with the entry of the ligand due to its high flexibility [130].
- SRS6 is the shortest region and is located in the loop near the *β*3 sheet [126].

The human CYP1A1 structure allows binding planar molecule with ~12.3 Å in length and ~4.6 Å in width, conformed by aromatic, polyaromatic, and heterocyclic rings which are essential for the formation of *π*-*π* stacking in the protein active site, mainly with Phe-224 at helix F, conferring stability to the enzyme-substrate complex [43, 80, 81, 131–135]. Nevertheless, for specific substrate redox reaction to be produced (Table 3), ligand also requires to be oriented with its reactive group facing the heme group [136, 137].

## 7. CYP1A1 through Development

Besides its importance in the metabolism of xenobiotics, CYP1A1 is also involved in the metabolism of endogenous compounds, such as arachidonic acid, eicosapentaenoic acid [93], 17*β*-estradiol [95], and melatonin [94].

Arachidonic acid and eicosapentaenoic acid are biotransformed by this enzyme to products such as 14, 15-epoxyeicosatrienoic acid and 17, 18-epoxyeicosatetraenoic acid, which influence cardiovascular pressure [93]. This attribute highlighted the importance of the association between heart diseases and CYP1A1 polymorphisms [138–140].

Treatment with the CYP1A inhibitor, *α*-naphthoflavone, shows that the activity of CYP1A1 is important for the proper development of the embryo's cardiovascular system [141–143]. However, so far there is not enough information about the impact of this isoform in the endogenous metabolism, so it is essential to conduct more studies that can help us to understand the mechanisms of these processes and their impact on the human health.

The use of different animal models has proved that activity and basal expression of CYP1A1 during embryonic development are organ-stage-specific (Table 4), where the liver and cardiovascular tissues have the highest expression. In the chicken, exposure to CYP1A1 inducers causes an increase in heart size and weight, while, in fish, edema in pericardium as well as modifications in the normal shape of the organ has been reported [141, 142, 144–148].

Searching whether the function of CYP1A1 is crucial for life, a line of knockout mice for this gene was produced [149]. These animals show decreased liver, kidney, and heart weight, as well as increased blood pressure and lower heart rate compared to wild type mice, thus demonstrating the importance of CYP1A1 in the cardiovascular system [150].

In adulthood, the human CYP1A1 expression is low and is found particularly in tissues of the respiratory system such as trachea and lungs, but after induction, it is also detected in other organs such as liver, adrenal gland, bladder, heart, kidney, ovary, placenta, prostate, testis, thyroid, salivary gland, and spleen [96, 151]. Among these organs, different levels of the protein are detected [152].

## 8. Concluding Remarks

CYP1A1 is a relevant enzyme for biotransformation of environmental compounds into mutagenic metabolites; this fact has a strong effect on worldwide population; therefore, the knowledge of its tridimensional structure as well as its ligands allows us to the rationale search and development of inhibitors that would become chemopreventive agents for diseases related to exposure to CYP1A1 activated carcinogens.

On the other hand, the presence of CYP1A1 among several species forces us to choose biological models that share with humans similar CYP1A1 characteristics in order to obtain results able to be extrapolated. The animals frequently used for this purpose are rats and mice, in which some of the regulatory mechanisms and other data, reported here, have been described. Moreover, as already mentioned in the “upregulation of CYP1A1” Section, several pathways could be involved like the recently reported WNT-*β* catenin, RAR, or CAR pathways that regulate* CYP1A1* expression by direct interaction with its gene promoter or with that of* AHR* or both. However, these alternative pathways are poorly described and more studies in this regard are required to know how and what are the factors involved as well as the specific conditions necessary for their action on* CYP1A1* expression, like the tissue and its microenvironment or culture cell type used just to mention two of them. The discoveries of pathways that converge in CYP1A1 regulation are opportunities for the selection of new therapeutic targets that allow drug development for chemoprevention.

For the study of CYP1A1, we need to take into account that impairment of gene expression or enzyme activity could lead to adverse effects because it is involved in endogenous metabolism, an issue discussed in “CYP1A1 through development,” with particular interest in cardiotoxicity.

The integration of data generated about CYP1A1, factors, and mechanisms that play a role in carcinogen bioactivation will help us to rise up strategies that improve our life quality. In this context, some key questions that need to be addressed are written below.

It will be worth to continue the searching for chemopreventive agents that inhibit CYP1A1 even if it seems to be involved in the normal development of the heart. It is a good strategy to improve chemopreventive agents acting on different regulating CYP1A1 pathways at the same time; meanwhile they have fewer side effects. What is the real contribution of CYP1A1 in the process of carcinogen bioactivation knowing that it shares regulatory elements with additional CYPs of the same family? Do the cardiotoxicity effects produced in the lack of CYP1A1 activity be a window for searching new therapeutic targets for cardiovascular diseases? What is the biological relevance of reactive oxygen species production by CYP1A1? Why do tissues have differences on CYP1A1 expression? Is the tissue-specific, or even cell-specific, expression of CYP1A1 explained by differences in endogenous metabolism requirements or by alternative modulation of a particular set of AHR co-activators? Do the specific CYP1A1 expression and induction play a role in the development of a particular cancer ligand related?

## Figures and Tables

**Table 1:** Effect of DNA methyltransferases and histone deacetylases inhibition on CYP1A1 expression.

DNMT inhibitor dosing schedule	Cell type or specie	PAH type	Effect	DNA methylation status	Source
	Human cell adenocarcinoma, A549 Human bronchial epithelium cell line, Beas-2B	BaP 1 nM, 100 nM, and 10 uM	*hCYP1A1* expression started with 10 *μ*M. *hCYP1A1* expression started with 100 nM.	35% complete methylation 11% complete methylation.	[65]

5AzadC, 5 uM, 96 h	Human breast carcinoma cell line, MCF-7 Human cervical adenocarcinoma cell line, HeLa	10 nM TCDD lasts 24 hours	*hCYP1A1* expression increased 2-3-fold in Aza versus ctrl but did not change in Aza-TCDD versus TCDD. *hCYP1A1 *expression increased 4-fold in Aza versus ctrl and 7-fold in Aza-TCDD versus ctrl.	Both cell lines: highly methylated at CpG sites in enhancer region. Low methylated at CpG sites in promoter region.	[66]

5AzadC, 0, 0.25, and 1 uM	Human prostatic epithelial cell line, PWR1-E Human prostatic epithelial cell line, RWPE-1	TCDD, 10 nM	*hCYP1A1* expression increased in both PWR1 and RWPE1 treated with AzadC but not in the induction by TCDD.	RWP1 low methylated than LNCaP at enhancer region. No methylation at promoter.	[27]
	Human prostate adenocarcinoma cell line, LNCaP		LNCaP increased their *hCYP1A1 *induction by TCDD in a dose dependence of AzadC		

5AzadC, 2 uM, 72 h (each 12 h)	Mouse hepatoma cell line, Hepa1c1c7	5 uM BaP, 8 h	Aza does not change *mCYP1A1* expression versus control Aza-BaP does not change *mCyp1a1* induction versus BaP	ND	[28]

5AzadC, 5 uM, 3 days	Mouse hepatoma cell line, Hepa1c1c7 Mouse embryonic fibroblast, C3H10T1/2	10 nM TCDD, 48 h	Nonincrease *mCyp1a1* expression in Aza-TCDD induced versus TCDD. C3H10T1: *mCyp1a1* expression increased in Aza-TCDD induced versus TCDD.	ND	[67]

5AzadC, 5 uM, 72 h	Human breast cancer cell line, MCF7 Human hepatic cancer cell line, HepG2	TCDD last 24 h of 5AzadC treatment	MCF7, no differences. HepaG2. no differences.	ND	[29]

5AzadC 1, 5, 10, 50, 250, and 500 uM, 72 hours after EGF treatment	Primary rat hepatocytes (Sprague-Dawley rats)		rCYP1A1 protein increases in dose dependence of AzadC	ND	[68]

5AzadC, 0.5 uM, 5 days	Primary normal human bronchial epithelial cells, NHBE (*n* = 12). Human bronchial epithelial cell lines (HBEC *n* = 3). Human lung adenocarcinoma cell lines (HLAC *n* = 9)		AzadC increased *hCYP1A1* expression in HLAC	NHBE and HBEC were low methylated than HLAC at enhancer region.	[69]

5AzadC, 5 uM, 7 days (with culture media changed on day four). On day 6 cells were split into 60 mm dishes in culture media with AzadC. Day 7, media were changed.	Human cervical adenocarcinoma cell line, HeLa		AzadC increased *hCYP1A1* expression versus ctrl.	HeLa and HepG2 were equally methylated at promoter.	[70]

5AzadC, 5 *μ*M, 5 days 5 *μ*M RG108, 5 days	Human primary hepatocytes (hPH) Human embryonic stem cells derived hepatocytes (hESC.Hep)		hESC-Hep: increased *hCYP1A1* expression in both 5AzadC and RG108 treatments.	hPH: no methylated hESC-Hep: high methylated.	[71]

HDAC inhibitor dosing schedule	Cell line type	AHR ligand	Effect		Source

TSA (200 ng/mL), 30 min prior to TCDD	Mouse hepatoma cell line, Hepa1c1c7	TCDD, 1 pM	No effect on EROD basal enzyme activity Increased TCDD, concentration dependence induction of EROD enzyme activity and CYP1A1 protein		[72]

TSA, 100 ng/mL, 24 h	Human breast carcinoma cell line, MCF-7 Human cervical adenocarcinoma cell line, HeLa	TCDD 10 nM (after TSA), 24 h	Increased basal *hCYP1A1 *expression, but TSA had no effect on TCDD induced mRNA. Increased basal and TCDD induced *hCYP1A1 *mRNA		[66]

SAHA (0.2–4.0 *μ*M),12 and 24 h TSA (0.2–4.0 *μ*M), 12 and 24 h	Human breast carcinoma cell line, MCF-7	BaP, 4 *μ*M	Increased BaP induced EROD activity and basal *hCYP1A1 *mRNA No effects on BaP induced *hCYP1A1* mRNA Increased BaP induced EROD activity and basal *hCYP1A1 *mRNA Decreased BaP induced *hCYP1A1 *mRNA		[73]

TSA (25 *μ*M), 2, 4, and 7 days	Primary rat hepatocytes (Sprague Dawley)	None	Increased EROD activity at day 7. Increased rCYP1A1 protein at all days tested. Increased *rCYP1A1 *mRNA at days 4 and 7.		[74]

Sodium butyrate (NaB), 2 mM,16 h	Mouse hepatoma cell line, Hepa1c1c7	BaP, 5 *μ*M, 8 h	No changes on basal and induced m*Cyp1a1 *mRNA		[28]

TSA, 100 nM, 24 h	Mouse hepatoma cell line, Hepa1-OT Mouse embryonic fibroblast cell line, C3H10T1/2	TCDD, 10 nM, 24 h	Increased TCDD induced *mCyp1a1* mRNA Increased TCDD induced *mCyp1a1 *mRNA		[67]

AN-8 (1–5 *μ*M), 72 h	Primary hepatocytes culture	None	Increased CYP1A1 protein level		[68]

TSA 250 nM,16 h	Human cervical adenocarcinoma cell line, HeLa	PCB, 136 3 *μ*M (after TSA), 6 h	Increased basal and PCB induced *hCYP1A1* mRNA		[70]

ND: nondetermined. All increases or decreases in DNA methylation, mRNA, or protein were significantly different with respect to the respective control. For more information about this, references to the original work are provided.

EROD: Ethoxyresorufin *O-*deethylation CYP1A1 enzyme activity.

**Table 2:** Effect of mutations in the amino acid sequence of human CYP1A1 on the kinetic parameters of this enzyme.

Amino acid	Position	Amino acid type	Mutation	Amino acid type	Effect	Reference
Gly	45 loop A′	Nonpolar, aliphatic	Asp	Negatively charged	*K*_*m*_ and *V*_max⁡_ are decreased by 42.9% and 75.1%, respectively	[75]

Ala	62 helix A	Nonpolar, aliphatic	Pro	Nonpolar, aliphatic	*K*_*m*_ is increased by 84% and *V*_max⁡_ is decreased by 21%	[76]

Ser	116 helix B′	Polar, uncharged	Ala	Nonpolar, aliphatic	*K*_*m*_ and *V*_max⁡_ do not change	[77]

Ser	122 loop B′-C	Polar, uncharged	Thr	Polar, uncharged	Activity is increased by 25%	[78]
			Ala	Nonpolar, aliphatic	*K*_*m*_ and *V*_max⁡_ are increased by 74% and 2-fold, respectively	[79]

Phe	123 loop B′-C	Aromatic	Ala	Nonpolar, aliphatic	Without activity. *K*_*m*_ is increased by 12.8-fold and *V*_max⁡_ is decreased by 42.5%	[77, 79]

Glu	161 helix D	Negatively charged	Lys	Positively charged	*K*_*m*_ is decreased by 39% and *V*_max⁡_ does not change	[77]

Glu	166 helix D	Negatively charged	Gln	Nonpolar, aliphatic	*K*_*m*_ and *V*_max⁡_ are increased by 3.7-fold and 24%, respectively	[77]

Val	191 helix E	Nonpolar, aliphatic	Met	Polar, uncharged	*K*_*m*_ and *V*_max⁡_ do not change	[77]

Asn	221 helix F	Nonpolar, aliphatic	Thr	Polar, uncharged	Activity is decreased to 28%	[78]

Phe	224 helix F	Aromatic	Ala	Nonpolar, aliphatic	*V*_max⁡_ and *K*_*m*_ are decreased by 11.4-fold and 75%, respectively	[79]

Gly	225 helix F	Nonpolar, aliphatic	Val	Nonpolar, aliphatic	Activity is decreased to 19%	[78]

Val	228 helix F	Nonpolar, aliphatic	Thr	Polar, uncharged	*K*_*m*_ and *V*_max⁡_ do not change	[77]

Glu	256 helix G	Negatively charged	Lys	Positively charged	*K*_*m*_ is decreased by 70% and *V*_max⁡_ does not change	[77]

Tyr	259 helix G	Aromatic	Phe	Aromatic	*K*_*m*_ is increased by 2.7-fold and *V*_max⁡_ does not change	[77]

Asn	309 helix H	Nonpolar, aliphatic	Thr	Polar, uncharged	*K*_*m*_ and *V*_max⁡_ do not change	[77]

Leu	312 helix I	Nonpolar, aliphatic	Asn	Nonpolar, aliphatic	Activity is decreased to 42%	[78]
			Phe	Aromatic	*K*_*m*_ is increased by 89% and *V*_max⁡_ does not change	[77]

Asp	313 helix I	Negatively charged	Ala	Nonpolar, aliphatic	*K*_*m*_ and *V*_max⁡_ are increased by 21-fold and 28%, respectively	[77]
			Asn	Nonpolar, aliphatic	*K*_*m*_ is increased by 24.5-fold and *V*_max⁡_ is decreased by 37.5%	[77]

Gly	316 helix I	Nonpolar, aliphatic	Val	Nonpolar, aliphatic	*K*_*m*_ is increased by 17-fold and *V*_max⁡_ is decreased by 30%	[77]

Ala	317 helix I	Nonpolar, aliphatic	Tyr	Aromatic	Without activity	[79]
			Gly	Nonpolar, aliphatic	*K*_*m*_ is increased by 30-fold and *V*_max⁡_ is decreased by 25%	[77]

Asp	320 helix I	Negatively charged	Ala	Nonpolar, aliphatic	*K*_*m*_ is increased by 2.7-fold and *V*_max⁡_ is decreased by 35%	[77]

Thr	321 helix I	Polar, uncharged	Gly	Nonpolar, aliphatic	*K*_*m*_ is increased by 30% and *V*_max⁡_ is decreased by 70%	[79]
			Pro	Nonpolar, aliphatic	*K*_*m*_ is increased by 6.2-fold and *V*_max⁡_ does not change	[77]
			Ser	Polar, uncharged	*K*_*m*_ and *V*_max⁡_ are increased by 7.6-fold and 2-fold, respectively	[77]

Val	322 helix I	Nonpolar, aliphatic	Ala	Nonpolar, aliphatic	*K*_*m*_ is increased by 67% and *V*_max⁡_ does not change	[77]

Val	382 helix K/ loop *β*1–4	Nonpolar, aliphatic	Ala	Nonpolar, aliphatic	Activity is decreased to 66%	[78]
			Leu	Nonpolar, aliphatic	Activity is decreased to 7%	[78]

Ile	386 helix K/ loop *β*1–4	Nonpolar, aliphatic	Gly	Nonpolar, aliphatic	Without activity	[79]
			Val	Nonpolar, aliphatic	*K*_*m*_ and *V*_max⁡_ are increased by 87% and 58%, respectively	[77]

Ile	458 helix L	Nonpolar, aliphatic	Pro	Nonpolar, aliphatic	*K*_*m*_ is increased by 44% and *V*_max⁡_ does not change	[77]
			Val	Nonpolar, aliphatic	*K*_*m*_ and *V*_max⁡_ are decreased by 55% and 21%, respectively	[77]

Thr	497 loop *β*4	Polar, uncharged	Ser	Polar, uncharged	*K*_*m*_ is increased by 3-fold and *V*_max⁡_ does not change	[77]

**Table 3:** Reactions carried out by the human CYP1A1 depending on the type of substrate.

Origin	Category compound	Type of reaction	Source
Synthetic compounds	Polycyclic aromatic hydrocarbons	Oxidation Epoxidation	[80, 81]
	Nitrosamides	Nitroreduction	[82, 83]
	Arylamines	*N*-hydroxylation Oxidation	[80, 81]
	Benzotriazole	Oxidation	[84]
	Heterocyclic amines	*N*-hydroxylation Oxidation	[80, 81]
	Nitroarenes	Nitroreduction	[85]
	Azoaromatic amines	Oxidation	[80, 81]

Natural compounds	Difuranocumarin	Epoxidation Oxidation	[86]
	Nefrotoxin	Hydroxylation	[87]
	Flavonoid	Hydroxylation *O*-demethylation	[88, 89]

Drugs	Ellipticin	Oxidation	[90]
	Omeprazol	ND	[91]
	Oltipraz	ND	[92]

Endogenous substrates	Arachidonic acid	Hydroxylation	[93]
	Melatonin	Hydroxylation	[94]
	Eicosapentaenoic acid	Epoxidation	[93]
	Stradiol	Hydroxylation	[95]

**Table 4:** Basal expression and activity of CYP1A1 in different animal models.

Animal model	Development stage	Spatial localization	Detection method	Reference
Human	16–36 gestation weeks	Not determined-	PCR	[96]
	50–60 gestation weeks	Hepatic tissue	BZROD (microsomes) (8.8 ± 2.1 pmol/mg of protein/min^−1^)	[97]
	74–145 gestation days	Day 87: kidney Days 55, 70, 101, and 112: lung Days 45, 70, and 85: liver	PCR: southern blot	[98]

Mouse	E17	Not determined-	PCR	[96, 99]
	E7-E14	E7: extraembryonic ectoderm and mesoderm E8.5: myocardial cells in ventricular chamber E10: left and right heart ventricle Dorsal aorta and neuroepithelial cells of midbrain E12: myocardial cells of both heart ventricles and midbrain E13: dorsal aorta, heart, and epithelium of midbrain E14: dorsal aorta, both heart ventricles, and atrium Epithelium of midbrain and trigeminal ganglion.	*lacZ* reporter with the promoter of *CYP1A1*	[100]

Rat	15–29 gestation days	D15: liver D29: lung and liver	PCR Southern blot	[98]

Chicken	4–15 incubation days	D4–D7: embryonic pool D9–D15: liver D4–D15: yolk sac	EROD (microsomes) (<1 pmol/mg of protein/min^−1^) (>300 <1100 pmol/mg of protein/min^−1^) (>20 <400 pmol/mg of protein/min^−1^)	[101]
	17 incubation days	Liver	Run-on transcription assay	[102]
	18 incubation days	Liver kidney	EROD (microsomes) (35 ± 6 pmol/mg of protein/min^−1^) (25 ± 9 pmol/mg of protein/min^−1^)	[103]
	10 incubation days	Liver	q-PCR	[104]

Zebra Fish	8–128 hours after fertilization (hpf)	8 hpf: germs layers 32–80 hpf: cardiovascular system 104–128 hpf: cardiovascular system, liver, intestine, urinary tract, and kidney	EROD in vivo (>0.08 <0.5 pmol/mg of protein/min^−1^)	[105]
	48–120 hpf	Embryonic pool	q-PCR EROD in vivo (0.0107–0.0184 pmol/mg of protein/min^−1^)	[106]
	4–8 days after fertilization	Not determined	EROD in vivo (50–100 fmol h^−1^ larva^−1^)	[107]

Medaka fish	8 hpf	Not determined	EROD in vivo (arbitrary units)	[108]
	50–245 hpf	Gallbladder	EROD in vivo (arbitrary units)	[109]
